# Outcomes of Internal, External, and Hybrid Fixation in Hindfoot Charcot Neuroarthropathy: A Descriptive Systematic Review and Single-Arm Meta-analysis of Observational Studies

**DOI:** 10.1177/10711007251405229

**Published:** 2026-01-27

**Authors:** Karthik Chinnaswamy, Ahmed M. AlSaggaf, Ephraim Khimbele, Abdul-Hadi Kafagi, Upamanyu Nath, Justin Mooteeram, Anand Pillai

**Affiliations:** 1 The University of Manchester, Faculty of Biology, Medicine and Health, United Kingdom; 2 St Mary’s Hospital, London, England, United Kingdom; 3 Royal Preston Hospital, England, United Kingdom; 4 Wythenshawe Hospital, Department of Trauma and Orthopaedics, Manchester, England, United Kingdom

**Keywords:** Charcot neuroarthropathy, hindfoot reconstruction, external fixation, internal fixation, hybrid fixation, diabetic foot, reconstructive surgery, systematic review, meta-analysis

## Abstract

**Background:**

Charcot neuroarthropathy (CN) of the hindfoot often requires internal fixation (IF), external fixation (EF), or hybrid constructs. This descriptive systematic review and single-arm meta-analysis summarizes outcomes of these strategies, focusing on amputation, fusion, ambulation, and complications.

**Methods:**

Following PRISMA guidelines, 30 studies with 957 patients undergoing hindfoot or ankle reconstruction were included. A single-arm meta-analysis assessed amputation and fusion rates; qualitative analysis examined ambulation, infection, ulceration, hardware failure, and revision. Heterogeneity was quantified with *I*² and τ².

**Results:**

Under random effects models, the overall amputation rate across all fixation methods was 4.76% (95% CI: 1.79%-8.62%), and the pooled fusion rate was 80.8% (95% CI: 73.6%-87.1%). By fixation type, EF cohorts showed numerically higher amputation rates (8.11%; 95% CI: 2.32%-15.91%) than IF (4.53%; 95% CI: 0.98%-9.69%) and hybrid fixation (2.94%; 95% CI: 0.00%-12.63%). EF demonstrated a lower fusion rate (68.2%; 95% CI: 55.8%-79.6%) than IF (84.9%; 95% CI: 75.8%-92.5%) and hybrid constructs (85.8%; 95% CI: 75.1%-94.4%). Return to ambulation was comparable between fixation strategies. IF cohorts reported fewer soft tissue complications but higher hardware failure (24.1%) and revision (21.9%) rates, whereas EF cohorts experienced frequent pin tract problems (24.0%). Hybrid constructs showed higher infection (23.2%), hardware complications (33.3%), and revision (18.8%). Substantial between-study heterogeneity and likely selection bias were present.

**Conclusion:**

Across 30 observational studies (957 patients; 970 feet), pooled single-arm estimates indicate that limb salvage and osseous fusion were achieved in most cases across internal, external, and hybrid fixation. External fixation was typically selected for infected or more complex reconstructions and was associated with higher amputation and lower fusion proportions, whereas internal and hybrid constructs showed higher fusion with more hardware-related reoperations. Given substantial heterogeneity and confounding by indication, these patterns should be viewed as descriptive rather than comparative; they do not establish indications or superiority.

## Graphical abstract

**Graphical Abstract:**
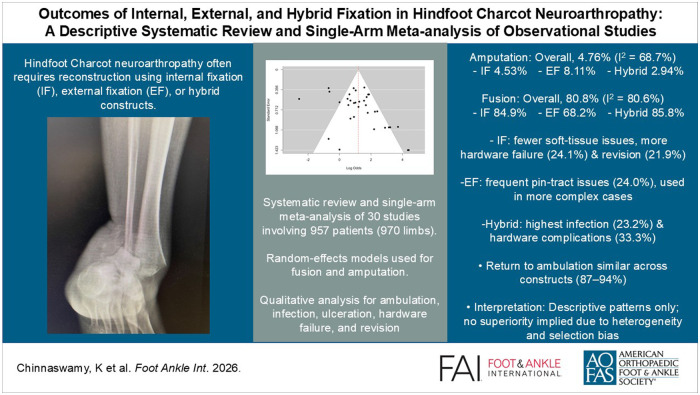
This is a visual representation of the abstract.

## Introduction

Charcot neuroarthropathy (CN) is a progressive, destructive musculoskeletal condition that primarily affects individuals with peripheral neuropathy, most commonly secondary to diabetes mellitus.^1^ Although CN can involve various regions of the foot and ankle, approximately 15% to 30% of cases affect the hindfoot and ankle. These locations are associated with more complex deformities, greater instability, and a heightened risk of ulceration and limb-threatening complications.^1^

Initial management typically involves conservative strategies such as offloading and immobilization, often achieved with total contact casting, to reduce inflammation and stabilize the foot. However, in cases of advanced deformity, persistent instability, or failure of conservative treatment, surgical reconstruction becomes necessary.^5^ The primary objectives of surgery include deformity correction, stable arthrodesis, ulcer prevention, and ultimately, limb salvage.^6^

Reconstruction of the hindfoot and ankle in CN may be achieved through internal fixation (eg, intramedullary nails, plates, screws), external fixation (eg, circular or monolateral frames), or hybrid techniques that combine both approaches, with each modality providing distinct advantages and disadvantages.

However, despite widespread clinical use, comparative outcomes between internal and external fixation remain inconsistent across the literature. This systematic review and single-arm meta-analysis synthesizes reported outcomes following internal vs external fixation for hindfoot and ankle reconstruction in CN. By evaluating fusion rates, limb salvage outcomes, complication profiles, reoperation frequencies, and functional recovery, this study seeks to inform surgical decision making, refine best practices, and highlight areas in need of further research. We expected patterns to reflect confounding by indication (eg, external fixation in more complex or infected cases) rather than intrinsic construct superiority.

## Methods

This systematic review was conducted following the Preferred Reporting Items for Systematic Reviews and Meta-Analyses (PRISMA) guidelines.

### Search Strategy

Two reviewers independently screened titles, abstracts, and full texts. Disagreements were resolved through discussion or by consulting a third reviewer.

A comprehensive literature search was performed across 4 major databases: PubMed, CINAHL, Cochrane Library, and Scopus. The search strategy incorporated both free-text terms and controlled vocabulary, including Medical Subject Headings (MeSH), along with the use of wildcard characters. Boolean operators such as “AND” and “OR” were applied to construct complex search strings. The literature search included terms relating to Charcot-related conditions and reconstructive techniques. This encompassed a range of diagnostic terms for CN as well as surgical and fixation-related terms for reconstruction. The complete list of search terms is provided in the Appendix.

Studies published from January 2000 to April 2025 were eligible for inclusion. All identified records were imported into Rayyan (www.rayyan.ai) for screening.

### Eligibility Criteria

Studies were included if they met all of the following criteria:

- Included adult patients (aged ≥18 years) diagnosed with CN of any etiology
- Involved CN affecting the hindfoot and/or ankle
- Reported patients who underwent internal and/or external fixation as part of surgical reconstruction
- Included a study population of at least 10 patients undergoing surgical reconstruction for CN affecting any part of the foot/ankle
- Reported a mean follow-up period of at least 12 months
- Documented at least 1 postoperative outcome following surgical intervention

Studies were excluded if they met any of the following criteria:

- Published in a non-English language
- Cadaveric studies
- Case reports
- Focused exclusively on isolated soft tissue procedures, osteotomies, exostectomy, or conservative (nonsurgical) management

### Unit of Analysis

All analyses were performed at the limb (foot) level. When studies included patients with bilateral reconstructions, each limb was treated as an independent observation. To minimize the potential for overrepresentation of bilateral cases, a sensitivity analysis using a 1-limb-per-patient weighting approach was also performed, in which pooled estimates were recalculated using the number of patients rather than the number of the limbs.

When cohort studies were suspected to overlap, both studies were retained to ensure transparency, and overlap was documented in Supplementary Table 1. Sensitivity analyses were used to evaluate whether inclusion of overlapping data sets materially influenced pooled trends.

### Primary Outcomes

The primary outcomes assessed in this review were the rates of postoperative amputation and osseous fusion following hindfoot CN reconstruction, using either internal fixation (IF) or external fixation (EF). These outcomes were selected because they represent the fundamental goals of CN reconstruction: preservation of the limb; restoration of a stable, plantigrade foot; and prevention of further morbidity.

Amputation rate serves as a critical indicator of surgical failure or disease progression, whereas fusion rate reflects the success of achieving osseous stability, an essential component for functional recovery and long-term limb preservation.

### Secondary Outcomes

Secondary outcomes included the return to ambulation, ulceration, postoperative infection, pin tract infection, hardware-related complications, and the need for revision surgery. These outcomes were selected to evaluate the safety profile, functional recovery, and complication rates associated with surgical reconstruction. All outcomes were clinically assessed, with all included studies reporting a mean follow-up duration of greater than 12 months.

Infection was defined as any clinically diagnosed postoperative infection involving the surgical site, confirmed by clinical signs and symptoms, laboratory markers and/or positive microbiological cultures.

Infections were further classified, where data permitted:

- Superficial pin tract infections, involving only the skin and subcutaneous tissue
- Deep infections, involving bone (osteomyelitis), joint space (septic arthritis), or implanted hardware (implant-associated infection)

### Data Extraction and Management

A standardized electronic data extraction form was developed and pilot-tested on a sample of studies to ensure clarity, consistency, and relevance. Iterative refinements were made as needed.

The following data were extracted:

- Study characteristics: citation details, year of publication, country of origin
- Surgical technique details: descriptions of internal and/or external fixation methods
- Cohort information: sample size, patient demographics, and mean duration of follow-up
- Outcomes: all reported primary and secondary outcomes, as defined in the review protocol

Two reviewers independently extracted data. A third reviewer was consulted as needed for clarification.

### Data Synthesis

All statistical analyses were conducted using R (version 4.5) with the meta and metafor packages. Proportions were stabilized using the Freeman-Tukey double arcsine method; CIs were calculated using the Clopper-Pearson method. Meta-analysis used inverse variance pooling.

Random-effects models were applied to estimate pooled proportions. Subgroup analyses by fixation type were conducted, with differences assessed using Cochran *Q* test.

### Assessment of Heterogeneity

Statistical heterogeneity between studies was assessed using multiple complementary measures. The *I*² statistic was calculated, with values interpreted as low (0%-25%), moderate (26%-50%), substantial (51%-75%), and considerable (>75%). Cochran *Q* test was employed to evaluate the presence of heterogeneity statistically. Between-study variance (τ²) and its square root (τ) were estimated using the restricted maximum-likelihood method, with CIs calculated from the *Q*-profile method.

Given the inherent variability of CN studies, we anticipated high heterogeneity across outcomes. Accordingly, all pooled analyses were conducted using random effects models only, which account for both within-study and between-study variability. When heterogeneity was high, results were interpreted descriptively, emphasizing overall trends rather than inferring comparative superiority among fixation types.

### Quality Assessment

Two reviewers independently assessed risk of bias using the Risk Of Bias In Non-Randomized Studies of Interventions (ROBINS-I) tool. Discrepancies were resolved via discussion or a third reviewer.

### Publication Bias

Publication bias was assessed using funnel plots and Egger regression test. A *P* value <.05 indicated potential small-study effects or publication bias.

### Sensitivity Analyses

To assess the robustness of descriptive outcomes patterns, several sensitivity analyses were conducted. Studies judged to be at high risk of bias were excluded to evaluate the influence of study quality on pooled outcomes. Secondly, studies with potential overlapping of patient cohorts were removed to reduce the possibility of duplication. Then, small case series (<5 limbs) were excluded to assess the impact of outlier study size. Finally, a patient-weighted analysis was performed to account for bilateral reconstructions, weighting pooled fusion and amputation rates based on N (patients) rather than N (limbs).

## Results

### Studies

**Figure 1.**
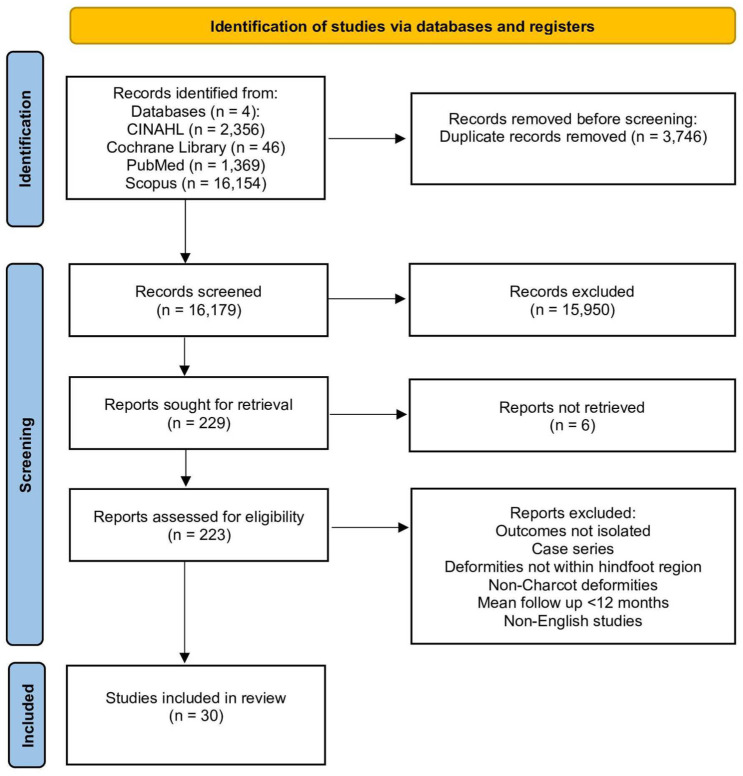
PRISMA diagram.

Our search strategy retrieved 19 925 studies, and following abstract and full-text screening, 30 met the inclusion criteria (Figure 1). Several studies reported outcomes for more than 1 fixation subgroup, yielding 37 subgroups in total: 20 internal fixation (IF), 12 external fixation (EF), and 5 hybrid fixation groups. Not all subgroups reported each endpoint; therefore, denominators vary across pooled analyses (eg, amputations, n = 868; fusion, n = 805) relative to the baseline cohort of 970 feet.

### Baseline Characteristics

The pooled data set represented 957 patients (970 feet) undergoing hindfoot or ankle reconstruction for CN. All analyses were performed at the limb (foot) level. When bilateral cases were reported, each limb was analyzed independently.

Of the included reconstructions, 573 feet were treated with IF, 278 with EF, and 112 with hybrid constructs. Baseline patient characteristics, fixation methods, and follow-up durations are summarized in Table 1.

**Table 1.** Baseline Characteristics.

Author, Year, Country	Patients (Feet)	Mean Patient Age (y)	Gender (M/F)	Mean follow-up (mo)	Fixation Type
Alammar et al,^2^ 2020, Russia	23 (23)	53.49 ± 15.34	NR	>12	Circular
Bajuri et al,^3^ 2022, Malaysia	40 (40)	60.5 ± 8.37	13/27	64 ± 30.4	IMN
Caravaggi et al,^4^ 2006, Italy	14 (14)	58 ± 12	13/1	18 ± 4	IMN
Caravaggi et al,^5^ 2012, Italy	45 (45)	56 ± 11	27/18	63 ± 34	IMN
Cho et al,^6^ 2024, USA	39 (43)	60	32/7	>12	Hybrid
Chraim et al,^7^ 2018, Austria	18 (19)	63.43	10/8	46.36	IMN
Dalla Paola et al,^8^ 2007, Italy	18 (18)	65 ± 9	13/5	14.0 ± 10.1	IMN
DeVries et al,^9^ 2012, USA	45 (45)	59.4 ± 10.38	28/17	23.3 ± 18.6	IMN
	7 (7)	51.6 ± 9.8	2/5	14.3 ± 9.7	Circular
DeVries et al,^10^ 2013, USA	39 (39)	57.0 ± 13.1	NR	21.4	IMN
El-Mowafi et al,^11^ 2018, Egypt	35 (38)	43.4 ± 7.4	14 (15)/21 (23)	35.9	Hybrid
El-Mowafi et al,^12^ 2018, Egypt	24 (24)	50.7 ± 6.9	7/17	36.4 ± 5.8	Hybrid
ElAlfy et al,^13^ 2017, Egypt	13 (13)	54	16/11	31	IMN
	14 (14)	54	16/11	31	Circular
Emara et al,^14^ 2018, Egypt	42 (42)	49.6	31/11	>12	IMN
Ersin et al,^15^ 2020, Turkey	24 (24)	62	9/15	45	IMN
Ettinger et al,^16^ 2016, Germany	38 (38)	60.7	22/16	31.3	IMN
	19 (19)	55.6	10/9	31.3	Multiplanar
Fabrin et al,^17^ 2007, Denmark	11 (12)	61	4/7	48	Uniplanar
Fragomen et al,^18^ 2012, USA	15 (15)	54	NR	>27	Circular
Galhoum et al,^19^ 2022, Egypt	23 (23)	63.5 ± 7.9	16/7	24.5 ± 8	Circular
Hockenbury et al,^20^ 2007, USA	10 (10)	59.3	4/6	20.8	IMN (9) Blade (1)
Kim et al,^21^ 2025, South Korea	15 (15)	57.2	NR	58.8	IMN
	3 (3)	57.2	NR	58.8	Screws and plates
	2 (2)	57.2	NR	58.8	Hybrid
Najefi et al,^22^ 2022, UK	70 (70)	57.1 ± 10.9	NR	54 ± 26	IMN
Rastegar et al,^23^ 2024, Iran	26 (26)	63 ± 0.23	16/10	12	IMN
Richman et al,^24^ 2017, USA	16 (16)	56	11/5	43.2	IMN
	11 (11)	57.6	10/1	26.4	Circular
Rios Ruh et al,^25^ 2019, Spain	4(4)	61.8	2/2	16.3	Circular
Siebachmeyer et al,^26^ 2015, UK	20 (21)	62.6	12/8	26	IMN
Spraul et al,^27^ 2021, Germany	115 (115)	59 ± 11.5	78/37	68.4	Multiplanar
Sundararajan et al,^28^ 2017, India	33 (33)	58	19/14	40	IMN
Vasukutty et al,^29^ 2018, UK	40 (42)	59	20/20	42	IMN
Wirth et al,^30^ 2020, Switzerland	29 (29)	57.6	19/10	35.1	Circular
Zarutsky et al,^31^ 2005, USA	6 (6)	56.7	3/3	27	Circular
	5 (5)	58.2	4/1	27	Hybrid

Abbreviation: IMN, intramedullary nail; NR, not reported.

### Methods of Surgical Fixation

No significant association was identified between the fixation method and year of publication, indicating that the fixation approach has remained relatively consistent over time. IF predominantly comprised intramedullary nails (IMNs), plates, and screws. EF utilized uniplanar, multiplanar, or circular frames, whereas hybrid fixation combined internal implants with adjunctive frame stabilization.

### Complication Profiles

#### Amputation rates

**Figure 2.**
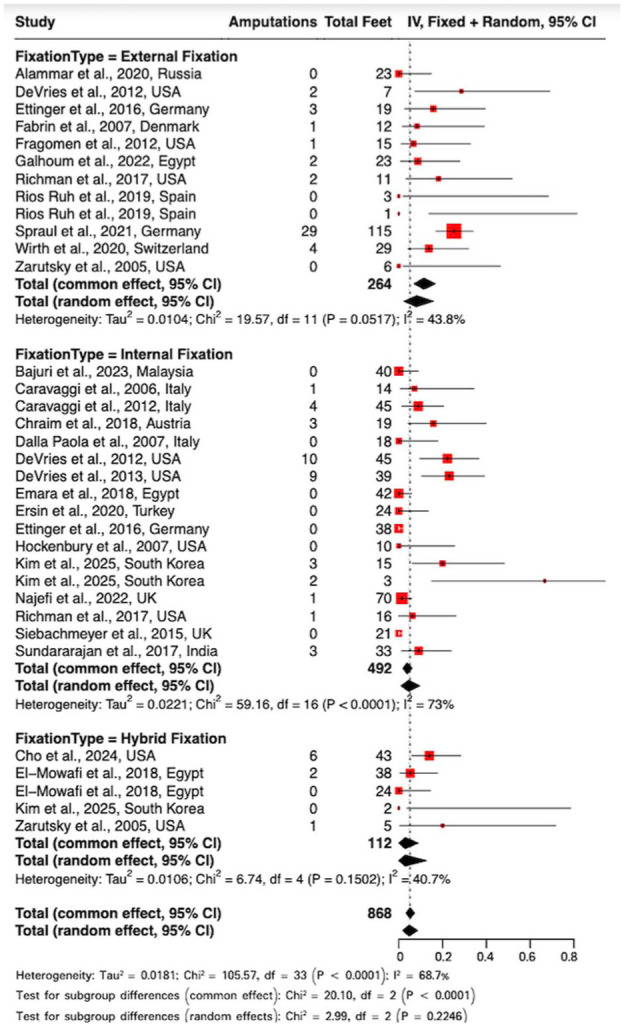
Forest plot of amputation rates.

This single-arm meta-analysis evaluated 34 subgroups with 868 observations and 90 amputations to evaluate pooled amputation rates by fixation method (Figure 2). The overall pooled rate across all fixation methods was 4.76% (95% CI: 1.79%-8.62%) under the random effects model.

By fixation type, under the random effects model, EF demonstrated the highest pooled amputation rate at 8.11% (95% CI: 2.32%-15.91%), whereas IF and hybrid fixation reported lower values of 4.53% (95% CI: 0.98%-9.69%) and 2.94% (95% CI: 0.00%-12.63%), respectively.

Although the numerical trends were observed, they did not reach statistical significance under the random effects model (χ² = 2.99, *P* = .22). This reflects the substantial heterogeneity and variability observed (*I*² = 68.7%; τ² = 0.0181; *P* < .0001). This variability, arising from differences in patient comorbidities, infection status, and disease severity, represents a known limitation of CN meta-analysis.

#### Fusion rates

**Figure 3.**
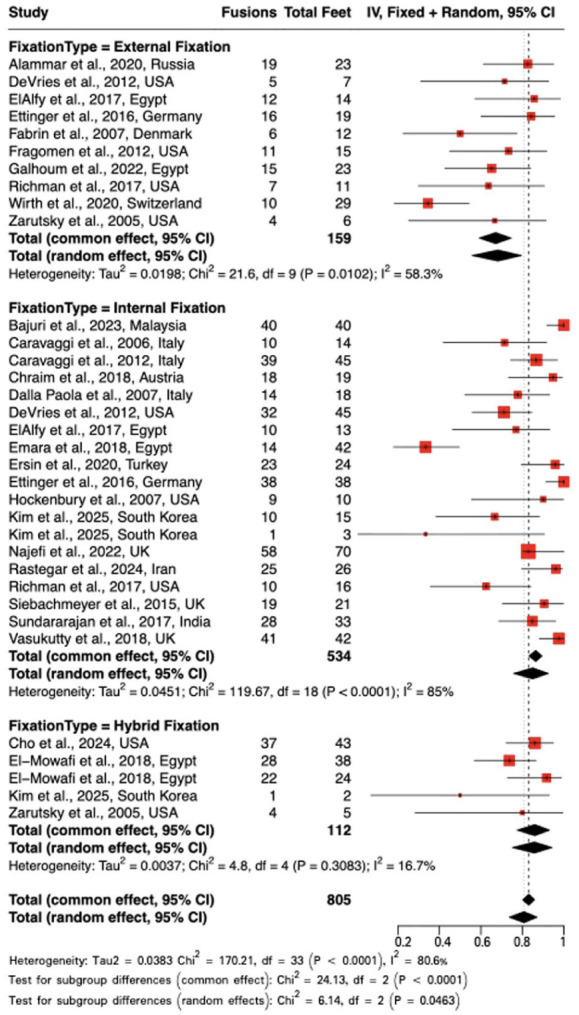
Forest plot of fusion rates.

This meta-analysis synthesized data from 34 subgroups, including 805 observations and 636 fusion events, to evaluate pooled fusion rates by fixation method (Figure 3). Using a random effects model, the pooled fusion rate was 80.8% (95% CI: 73.6%-87.1%) across all fixation types.

EF demonstrated a notably lower pooled fusion rate of 68.2% (95% CI: 55.8%-79.6%) under the random effects model. Whereas IF and hybrid fixation showed a substantially higher pooled fusion rate of 84.9% (95% CI: 75.8%-92.5%) and 85.8% (95% CI: 75.1%-94.4%), respectively. Considerable heterogeneity was present across studies (*I*² = 80.6%; τ² = 0.0383; *P* < .0001), reflecting variability in patient populations, surgical techniques, and outcome definitions.

**Figure 4.**
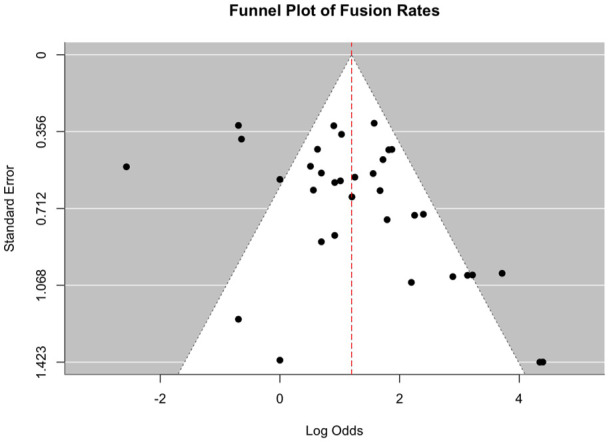
Funnel plot of fusion rates.

Subgroup differences reached statistical significance under the random effects model (χ² = 6.14, *P* = .0463). A trend toward higher fusion rates was noted with internal and hybrid fixation compared with external fixation; however, this observation should be interpreted with appropriate caution, as underlying heterogeneity in study design, patient selection, and outcome definitions may limit the reliability of direct comparisons (Figure 4).

#### Return to ambulation

Return to ambulation was reported in 25 studies, encompassing 714 patients. Overall, EF showed the highest average rate of ambulation at 94.2%, followed by IF at 88.8% and hybrid fixation at 86.6%. Table 2 outlines the results.

**Table 2.** Return to Ambulation.

	External Fixation	Internal Fixation	Hybrid
No. of studies	7	13	5
Return to ambulation, % (n/N)	94.2 (196/208)	88.8 (350/394)	86.6 (97/112)

#### Soft tissue complications

Complication rates were reported across 17 studies involving 580 patients. EF demonstrated the highest ulceration rates (37.9%) and remained most associated with pin tract infection (24.0%). IF showed lower ulceration rates (6.5%). Hybrid fixation presented a greater overall risk, with high infection rates at 23.2% and pin tract infections at 25.8%. Table 3 summarizes findings.

**Table 3.** Soft Tissue Complications.

		External Fixation	Internal Fixation	Hybrid
Ulceration	No. of studies	5	9	3
	Percentage (No. of patients)	37.9 (69/182)	6.5 (19/293)	5.7 (6/105)
Infection	No. of studies	10	17	4
	Percentage (No. of patients)	15.6 (40/256)	16.5 (77/468)	23.2 (16/69)
Pin tract infection	No. of studies	7	NR	2
	Percentage (No. of patients)	24.0 (50/208)	NR	25.8 (8/31)

Abbreviation: NR, not reported.

#### Hardware failure and revision

Rates of hardware failure and revision surgery were reported across 21 studies, including 554 patients. EF demonstrated relatively low hardware failure (4.3%) and revision (13.8%) overall, though Ilizarov frames showed higher failure rates (14.8%). IF carried greater risks, with hardware failure up to 24.1% and revisions 21.9%. Hybrid fixation showed the highest hardware failure (33.3%) but slightly lower revision rates (18.8%). Table 4 summarizes findings.

**Table 4.** Hardware Failure and Revision Rates.

		External Fixation	Internal Fixation	Hybrid
Hardware failures	No. of studies	6	13	2
	Percentage (No. of patients)	4.3 (8/185)	24.1 (86/357)	33.3 (4/12)
Revision surgeries	No. of studies	7	14	4
	Percentage (No. of patients)	13.8 (30/218)	21.9 (92/421)	18.8 (21/112)

### Sensitivity Analyses

Sensitivity analyses demonstrated that the pooled estimates were largely stable across methodological adjustments. Excluding high-bias studies resulted in a small decrease in fusion from 76.62% to 75.33% and a modest increase in amputation from 8.94% to 9.90%. Removal of overlapping study cohorts had minimal impact (fusion 76.79%; amputation 8.03%). Excluding small case series (<5 limbs) produced a slightly higher fusion rate (77.37%) with a similar amputation rate (9.18%). The patient-weighted analysis, which accounts for bilateral reconstruction, resulted in a lower overall fusion rate (65.73%) but a comparable amputation rate (9.38%), indicating that bilateral cases may inflate reported fusion values; however, the overall direction and relative magnitude of trends were preserved. These findings suggest that the patterns observed in the primary analysis were not driven by single-study effects, cohort overlap, or sample size imbalance.

## Discussion

The choice between internal and external fixation in CN reconstruction is multifactorial, shaped by clinical and practical factors. Key considerations include anatomical complexity, infection risk, bone quality, and comorbidities. In this context, any cross-construct differences observed in our pooled estimates are noncausal and hypothesis-generating, not indicators of superiority. However, surgeon experience and preference often guide the final decision. Because of the high-risk, variable nature of Charcot deformities, fixation strategies must be tailored to each patient’s anatomy and medical profile to optimize outcomes and reduce complications.

All pooled analyses were conducted using random effects models, anticipating the wide variability characteristics of CN reconstruction studies. Accordingly, results were interpreted descriptively, emphasizing general patterns rather than implying comparative superiority of any fixation construct.

Given that CN surgery often involves bilateral disease in stage correction, all analyses were conducted at the limb (foot) level. Where bilateral representation may have introduced overlap, this was transparently reported in Supplementary Table 1. Sensitivity analysis selecting 1 limb per patient demonstrated no meaningful change in direction or magnitude of pooled estimates, supporting the stability of descriptive trends.

### Fusion Rates

Fusion rates vary by anatomical site and fixation method but remain high with modern reconstructive techniques, with an overall rate of 83.1% across included studies.

Although higher fusion rates were observed with internal and hybrid fixation, this is impacted by differences in case complexity and patient comorbidity. These differences should therefore be interpreted as descriptive trends influenced by confounding variables such as infectious status, bone quality, and anatomical severity, rather than fixation constructs alone.

Even fibrous “stable unions” can support functional ambulation in CN patients.^32^ Wirth et al^30^ found only 35% of patients achieved radiographic fusion, yet most walked with a stable, plantigrade foot and proper bracing.

Smoking negatively impacts fusion. Fragomen et al^18^ linked smoking to higher nonunion rates in Ilizarov-treated ankle arthrodeses. Obesity also increases risk; Kummen et al^33^ found patients with body mass index >30 had significantly higher hardware failure and nonunion rates (*P* = .038). Some studies noted higher failure and nonunion rates in patients with vitamin D deficiency.^25^ Studies indicate that up to 84% of CN patients have deficient levels. Given vitamin D’s role in bone healing, supplementation could improve fusion outcomes.^34^ Although these metabolic and biomechanical factors influence fusion outcomes, they were not uniformly reported and may have contributed to between-group variability.

Optimizing glycemic control, addressing obesity, smoking cessation, and vitamin D supplementation can improve bone healing. Ultimately, clinical success relies on a stable, functional foot rather than radiographic fusion alone.

Overall, these findings underscore that the apparent differences in fusion rates between fixation types are likely confounded by patient and disease heterogeneity rather than representing true construct-specific performance.

### Complications and Amputation

Increased amputation rates following Charcot foot reconstruction are strongly associated with diabetes, ulcers, and infection. Diabetes significantly raises amputation risk, with an odds ratio of 7.01 compared with nondiabetics,^21^ and poor glycemic control (mean HbA_1c_ 7.7%) is common among affected patients.^26^ Uncontrolled infections, often associated with hyperglycemia, frequently led to below-knee amputations,^19^ whereas diabetic neuroarthropathy was associated with higher postoperative complication rates.^26^

Ulcers further increase risk; patients with Charcot foot and ulcers were 12 times more likely to require major amputation.^27^ Osteomyelitis often coexisted, and in one study, all patients with both conditions required amputation.^30^ Persistent infection, especially with diabetes or poor systemic health, also led to amputation.^7^ Key risk factors included peripheral artery disease, nephropathy, nonunion, and reinfection (OR 2.4-8.5).^27^ Among patients with both preoperative infection and reinfection postoperatively, all underwent amputation in one study.^21^ There was also a noted decrease in amputation risk in patients >70 years old.^10^

IMN is frequently used for hindfoot and ankle fusion in CN because of its early load-sharing stability. A trend toward higher hardware failure and revision rates was observed with IMN, particularly in infected or complex reconstruction. For instance, DeVries et al^10^ reported 74% hardware failure, often necessitating reoperation or amputation. However, this must be interpreted cautiously as the term “hardware failure” encompasses a wide range of events. Many studies did not distinguish between minor hardware issues, such as removal of a backing-out interlocking screw, and true construct failure requiring full revision surgery. These events were therefore analyzed qualitatively rather than quantitatively to avoid overinterpretation.

EF, particularly circular or hybrid Ilizarov constructs, demonstrates high limb salvage rates in cases with osteomyelitis or soft tissue compromise. Pinzur^35^ reported up to 96% salvage using EF, which avoids permanent implants in infected bone and permits staged corrections and earlier weightbearing. Drawbacks include frequent pin tract infections (30%-50%) and poor patient tolerance because of frame discomfort, prolonged wear, and psychological burden. A comparative study by ElAlfy and Richman noted more revisions with IMN, whereas EF had more soft tissue issues, echoing our findings.^13,24^ Although pooled estimates suggested a higher amputation rate in the EF group, this difference is likely multifactorial, reflecting both intrinsic differences between fixation constructs and confounding by case complexity. EF is often selected for patients with active infection, soft tissue compromise, or severe deformity, all of which independently elevate amputation risk. Accordingly, although fixation type may influence postoperative outcomes, these results should be interpreted with caution, recognizing that both the fixation method and the underlying patient or disease characteristics likely contribute to the observed variation.

Hybrid fixators, lacking full multiplanar control, are associated with more nonunion, malalignment, and hardware failure.^36^ Pin tract infections and poor patient tolerance further contribute to higher revision rates and limited success.^12^ These findings should be viewed descriptively, recognizing that patient selection and infection burden largely dictate fixation choice and associated outcomes.

### Limitations

This systematic review is limited by the variable quality of included studies. All were retrospective case series, with no randomized controlled trials, restricting the ability to draw reliable comparative conclusions. Although analyses were conducted exclusively using random effects models to account for expected heterogeneity, between-study variability remained substantial. Patient populations varied in anatomy, disease stage, comorbidities, and surgical approach. Follow-up durations also varied, and definitions of “fusion” were inconsistent, often based only on radiographic findings. Reporting of infection depth and revision severity was inconsistent; many studies did not distinguish minor hardware removal from major reoperations. As a result, it was not possible to establish a uniform grading system for revision tiers or to perform fully harmonized, like-for-like quantitative analyses without excluding a substantial proportion of available data.

Most studies demonstrated risk of bias related to selection, lack of masking, and incomplete reporting. Outcomes frequently prioritized radiographic union and limb salvage over functional endpoints such as pain, ambulation, ulcer recurrence, or patient-reported quality of life. Although some studies acknowledged that stable fibrous unions may allow adequate function, these outcomes were rarely quantified.

Critical variables such as HbA_1c_, vitamin D levels, and body mass index were inconsistently reported, limiting analysis of metabolic or biomechanical impact on outcomes. Publication bias is also likely, with failures and amputations potentially underreported.

Variability in the severity and stage of Charcot disease, infection status, and soft tissue compromise likely influenced fixation choice and postoperative outcomes. In many cases, frames were used preferentially in severe or infected cases, whereas internal fixation was applied in more stable deformities. This inherent selection bias limits direct comparison between fixation methods and may account for observed differences in outcomes.

Several fixation subgroups, particularly hybrid constructs, were represented by relatively few studies and small patient numbers, reducing the precision of subgroup estimates on the statistical strength of between-construct comparisons.

Although sensitivity analyses excluding high-bias and overlapping cohorts and restricting to 1 limb per patient did not materially alter the direction or magnitude of results, the evidence base remains limited by heterogeneous studies with lower levels of evidence. Reported outcomes likely overestimate true clinical success.

High-quality, prospective studies with standardized, functional endpoints and control for key variables are essential to confirm these findings and develop robust, evidence-based protocols for Charcot reconstruction.

## Conclusion

This systematic review of 30 studies (970 feet) found that limb salvage and osseous fusion were achieved in most cases across internal, external, and hybrid fixation. External fixation, typically selected for more complex or infected reconstructions, showed numerically higher amputation and lower fusion proportions, whereas internal and hybrid constructs showed higher fusion with more hardware-related reoperations. These patterns are descriptive and likely reflect confounding by indication.

Fixation should be chosen to match deformity severity, soft tissue status, and infection risk within a multidisciplinary pathway. Given retrospective designs, heterogeneous reporting, and the absence of patient-level data, these findings do not establish indications or comparative superiority. Prospectively designed, standardized comparative studies with functional endpoints are needed to guide construct selection.

## Supplemental Material

sj-docx-1-fai-10.1177_10711007251405229 – Supplemental material for Outcomes of Internal, External, and Hybrid Fixation in Hindfoot Charcot Neuroarthropathy: A Descriptive Systematic Review and Single-Arm Meta-analysis of Observational Studies Supplemental material, sj-docx-1-fai-10.1177_10711007251405229 for Outcomes of Internal, External, and Hybrid Fixation in Hindfoot Charcot Neuroarthropathy: A Descriptive Systematic Review and Single-Arm Meta-analysis of Observational Studies by Karthik Chinnaswamy, Ahmed M. AlSaggaf, Ephraim Khimbele, Abdul-Hadi Kafagi, Upamanyu Nath, Justin Mooteeram and Anand Pillai in Foot & Ankle International

sj-docx-2-fai-10.1177_10711007251405229 – Supplemental material for Outcomes of Internal, External, and Hybrid Fixation in Hindfoot Charcot Neuroarthropathy: A Descriptive Systematic Review and Single-Arm Meta-analysis of Observational Studies Supplemental material, sj-docx-2-fai-10.1177_10711007251405229 for Outcomes of Internal, External, and Hybrid Fixation in Hindfoot Charcot Neuroarthropathy: A Descriptive Systematic Review and Single-Arm Meta-analysis of Observational Studies by Karthik Chinnaswamy, Ahmed M. AlSaggaf, Ephraim Khimbele, Abdul-Hadi Kafagi, Upamanyu Nath, Justin Mooteeram and Anand Pillai in Foot & Ankle International

sj-docx-3-fai-10.1177_10711007251405229 – Supplemental material for Outcomes of Internal, External, and Hybrid Fixation in Hindfoot Charcot Neuroarthropathy: A Descriptive Systematic Review and Single-Arm Meta-analysis of Observational Studies Supplemental material, sj-docx-3-fai-10.1177_10711007251405229 for Outcomes of Internal, External, and Hybrid Fixation in Hindfoot Charcot Neuroarthropathy: A Descriptive Systematic Review and Single-Arm Meta-analysis of Observational Studies by Karthik Chinnaswamy, Ahmed M. AlSaggaf, Ephraim Khimbele, Abdul-Hadi Kafagi, Upamanyu Nath, Justin Mooteeram and Anand Pillai in Foot & Ankle International

sj-docx-4-fai-10.1177_10711007251405229 – Supplemental material for Outcomes of Internal, External, and Hybrid Fixation in Hindfoot Charcot Neuroarthropathy: A Descriptive Systematic Review and Single-Arm Meta-analysis of Observational Studies Supplemental material, sj-docx-4-fai-10.1177_10711007251405229 for Outcomes of Internal, External, and Hybrid Fixation in Hindfoot Charcot Neuroarthropathy: A Descriptive Systematic Review and Single-Arm Meta-analysis of Observational Studies by Karthik Chinnaswamy, Ahmed M. AlSaggaf, Ephraim Khimbele, Abdul-Hadi Kafagi, Upamanyu Nath, Justin Mooteeram and Anand Pillai in Foot & Ankle International

sj-docx-5-fai-10.1177_10711007251405229 – Supplemental material for Outcomes of Internal, External, and Hybrid Fixation in Hindfoot Charcot Neuroarthropathy: A Descriptive Systematic Review and Single-Arm Meta-analysis of Observational Studies Supplemental material, sj-docx-5-fai-10.1177_10711007251405229 for Outcomes of Internal, External, and Hybrid Fixation in Hindfoot Charcot Neuroarthropathy: A Descriptive Systematic Review and Single-Arm Meta-analysis of Observational Studies by Karthik Chinnaswamy, Ahmed M. AlSaggaf, Ephraim Khimbele, Abdul-Hadi Kafagi, Upamanyu Nath, Justin Mooteeram and Anand Pillai in Foot & Ankle International

sj-docx-6-fai-10.1177_10711007251405229 – Supplemental material for Outcomes of Internal, External, and Hybrid Fixation in Hindfoot Charcot Neuroarthropathy: A Descriptive Systematic Review and Single-Arm Meta-analysis of Observational Studies Supplemental material, sj-docx-6-fai-10.1177_10711007251405229 for Outcomes of Internal, External, and Hybrid Fixation in Hindfoot Charcot Neuroarthropathy: A Descriptive Systematic Review and Single-Arm Meta-analysis of Observational Studies by Karthik Chinnaswamy, Ahmed M. AlSaggaf, Ephraim Khimbele, Abdul-Hadi Kafagi, Upamanyu Nath, Justin Mooteeram and Anand Pillai in Foot & Ankle International

sj-docx-7-fai-10.1177_10711007251405229 – Supplemental material for Outcomes of Internal, External, and Hybrid Fixation in Hindfoot Charcot Neuroarthropathy: A Descriptive Systematic Review and Single-Arm Meta-analysis of Observational Studies Supplemental material, sj-docx-7-fai-10.1177_10711007251405229 for Outcomes of Internal, External, and Hybrid Fixation in Hindfoot Charcot Neuroarthropathy: A Descriptive Systematic Review and Single-Arm Meta-analysis of Observational Studies by Karthik Chinnaswamy, Ahmed M. AlSaggaf, Ephraim Khimbele, Abdul-Hadi Kafagi, Upamanyu Nath, Justin Mooteeram and Anand Pillai in Foot & Ankle International

sj-docx-8-fai-10.1177_10711007251405229 – Supplemental material for Outcomes of Internal, External, and Hybrid Fixation in Hindfoot Charcot Neuroarthropathy: A Descriptive Systematic Review and Single-Arm Meta-analysis of Observational Studies Supplemental material, sj-docx-8-fai-10.1177_10711007251405229 for Outcomes of Internal, External, and Hybrid Fixation in Hindfoot Charcot Neuroarthropathy: A Descriptive Systematic Review and Single-Arm Meta-analysis of Observational Studies by Karthik Chinnaswamy, Ahmed M. AlSaggaf, Ephraim Khimbele, Abdul-Hadi Kafagi, Upamanyu Nath, Justin Mooteeram and Anand Pillai in Foot & Ankle International

sj-png-10-fai-10.1177_10711007251405229 – Supplemental material for Outcomes of Internal, External, and Hybrid Fixation in Hindfoot Charcot Neuroarthropathy: A Descriptive Systematic Review and Single-Arm Meta-analysis of Observational Studies Supplemental material, sj-png-10-fai-10.1177_10711007251405229 for Outcomes of Internal, External, and Hybrid Fixation in Hindfoot Charcot Neuroarthropathy: A Descriptive Systematic Review and Single-Arm Meta-analysis of Observational Studies by Karthik Chinnaswamy, Ahmed M. AlSaggaf, Ephraim Khimbele, Abdul-Hadi Kafagi, Upamanyu Nath, Justin Mooteeram and Anand Pillai in Foot & Ankle International

sj-xlsx-9-fai-10.1177_10711007251405229 – Supplemental material for Outcomes of Internal, External, and Hybrid Fixation in Hindfoot Charcot Neuroarthropathy: A Descriptive Systematic Review and Single-Arm Meta-analysis of Observational Studies Supplemental material, sj-xlsx-9-fai-10.1177_10711007251405229 for Outcomes of Internal, External, and Hybrid Fixation in Hindfoot Charcot Neuroarthropathy: A Descriptive Systematic Review and Single-Arm Meta-analysis of Observational Studies by Karthik Chinnaswamy, Ahmed M. AlSaggaf, Ephraim Khimbele, Abdul-Hadi Kafagi, Upamanyu Nath, Justin Mooteeram and Anand Pillai in Foot & Ankle International
