# Supplementary material for: Outcomes of Internal, External, and Hybrid Fixation in Hindfoot Charcot Neuroarthropathy: A Descriptive Systematic Review and Single-Arm Meta-analysis of Observational Studies
Source: Foot Ankle Int. 2026 Jan 27;47(3):359–69. doi: 10.1177/10711007251405229 (PMC12966367; doi:10.1177/10711007251405229)
Supplement: sj-docx-4-fai-10.1177_10711007251405229 – Supplemental material for Outcomes of Internal, External, and Hybrid Fixation in Hindfoot Charcot Neuroarthropathy: A Descriptive Systematic Review and Single-Arm Meta-analysis of Observational Studies [file sj-docx-4-fai-10.1177_10711007251405229.docx]

**FAI and FAO Disclosure Form**

In order to promote complete transparency regarding relationships that could be considered a conflict of interest (COI), this FAI/FAO Disclosure Form must be **completed by each author** of this manuscript at the time of submission. If conflict of interest changes occur between submission and acceptance of the manuscript, an updated disclosure must be provided.

Please list any relationships with manufacturers or their related surrogate companies of devices, drugs or biologics. In doing so, consider this with a broad definition for relevant entities or relationships, and acknowledge any financial support for an author or close personal relation (including family members). Include anything within the **past 3 years** **of value above $500** that might be perceived to create bias.

**Types of conflicts of interest that must be disclosed:**

1. **Financial Relationships:** Such as receiving monetary benefits, funding, honoraria or other benefits from organizations that could be related to your research or publication.
2. **Professional Interests:** Authors should openly disclose any affiliations with organizations or institutions that could influence or be influenced by the results of their publication.

Authors and researchers are required to transparently disclose any affiliations or professional connections to organizations that might be impacted by the findings of their publication. Highlighting these affiliations is crucial for maintaining the transparency and credibility of their work.

*For instance, if your manuscript mentions a specific product or method involved in a procedure, and you benefit financially from it—such as receiving royalties, consultancy fees, or hospitality (including meals, travel, or accommodation) from the associated company—this must be disclosed for any cumulative financial payments exceeding $500 connected to that product or method.*

**FAI and FAO Disclosure Form**

| **Author Name:** | **Ephraim Khimbele** |
| --- | --- |
| **Manuscript Title:** | **Outcomes of Internal, External, and Hybrid Fixation in Hindfoot Charcot Neuroarthropathy: A Systematic Review and Single-Arm Meta-Analysis** |
| **Manuscript Number:** | **FAI-25-0638** |
| **Date:** | **27/08/2025** |

1. List any project funding, consulting fees, material support (including surgical supplies, cadavers, or the use of any products from related companies) or any other form of assistance valued over $500 received in the past three years.

Items relevant to this specific manuscript: specify type (consultancies, royalties, research support, other support, intellectual property etc.)

| N/A |
| --- |

2. Identify any additional financial or non-financial relationships from the past three years that could be perceived as conflicts of interest or could introduce bias in your submitted work (consultancies, royalties, research support, other support, intellectual property etc.)

| N/A |
| --- |

*This form will be available to the readership by publication as an online supplement to the article. Items relevant to this specific manuscript will be listed within the article text.*
