# Supplementary material for: Outcomes of Internal, External, and Hybrid Fixation in Hindfoot Charcot Neuroarthropathy: A Descriptive Systematic Review and Single-Arm Meta-analysis of Observational Studies
Source: Foot Ankle Int. 2026 Jan 27;47(3):359–69. doi: 10.1177/10711007251405229 (PMC12966367; doi:10.1177/10711007251405229)
Supplement: sj-docx-8-fai-10.1177_10711007251405229 – Supplemental material for Outcomes of Internal, External, and Hybrid Fixation in Hindfoot Charcot Neuroarthropathy: A Descriptive Systematic Review and Single-Arm Meta-analysis of Observational Studies [file sj-docx-8-fai-10.1177_10711007251405229.docx]

CN related terms included: “*Charcot neuroarthropathy”*, “*Charcot arthropathy”*, “*Charcot foot”*, “*Charcot*”, “neuropathic osteoarthropathy”*, and “*neuroarthropathy”*.

Reconstruction-related terms included: “*reconstructive surgery”*, “*reconstruction”*, “*surgical reconstruction”*, “*fixat*”*, “*limb salvage”*, “*surgery”*, “*arthrodesis”*, “*internal fixation”*, “*external fixation”*, “*Ilizarov”*, “*fusion”*, “*circular*”*, “*Taylor Spatial”*, “*hind foot nail”*, “*hind foot plate”*, “*mid foot plate”*, “*mid foot beams”*, and “*screws”*.
